# Efficacy and safety of immunosuppressive medications for steroid-resistant nephrotic syndrome in children: a systematic review and network meta-analysis

**DOI:** 10.18632/oncotarget.20377

**Published:** 2017-08-21

**Authors:** Shaojun Li, Haiping Yang, Pengfei Guo, Xiaoxiao Ao, Junli Wan, Qiu Li, Liping Tan

**Affiliations:** ^1^ Department of Emergency, Children's Hospital of Chongqing Medical University, Chongqing, China; ^2^ Department of Nephrology, Children's Hospital of Chongqing Medical University, Chongqing, China; ^3^ Ministry of Education Key Laboratory of Child Development and Disorders, Chongqing, China; ^4^ Key Laboratory of Pediatrics in Chongqing, Chongqing, China; ^5^ Chongqing International Science and Technology Cooperation Center for Child Development and Disorders, Chongqing, China

**Keywords:** immunosuppressant, SRNS, pediatrics, multiple-treatments meta-analysis

## Abstract

**Background:**

Conventional meta-analyses and randomized controlled trials have shown inconsistent results regarding the efficacy of immunosuppressants for pediatric steroid-resistant nephrotic syndrome (SRNS).

**Objective:**

To conduct a network meta-analysis aimed at evaluating the efficacy and safety of available immunosuppressive agents in pediatric patients with SRNS.

**Study methods:**

MEDLINE, the Cochrane Central Register of Controlled Trials, and EMBASE were searched on January 2017. Data from randomized controlled trials (RCTs) were included. The main outcomes analyzed were efficacy [number/portion with complete remission (CR), number/portion with partial remission (PR), and total number/portion in remission (TR)] and safety [adverse secondary event (ASE) rates].

**Results:**

A meta-analysis of 18 RCTs showed that tacrolimus was more efficacious for achieving CR than intravenous (i.v.) cyclophosphamide, mycophenolate mofetil (MMF), oral cyclophosphamide, leflunomide, chlorambucil, azathioprine, and plaebo/nontreatment (P/NT), and more efficacious than i.v. cyclophosphamide, oral cyclophosphamide, and P/NT in terms of TR outcomes. Cyclosporin was associated with a greater CR rate than i.v. cyclophosphamide, MMF, oral cyclophosphamide, chlorambucil, azathioprine, or P/NT, and associated with a greater TR rate than i.v. cyclophosphamide, oral cyclophosphamide, or P/NT. MMF was found to be more efficacious than i.v. cyclophosphamide and oral cyclophosphamide in terms of TR.

**Conclusions:**

Tacrolimus and cyclosporine may be preferred initial treatments for children with SRNS. MMF may be another option for this patient population. Further studies of the efficacy and safety of these three drugs in children with SRNS should be pursued.

## INTRODUCTION

The incidence of pediatric primary nephrotic syndrome (NS) is about 1–3/100,000 children 16 years old or younger [1]. In most cases, clinical remission of primary NS can be achieved with corticosteroid therapy [2]. The approximately 10–20% for whom complete remission is not achieved following corticosteroid therapy are classified as having steroid-resistant nephrotic syndrome (SRNS) [1]. SRNS patients are a heterogenous population with related diagnoses of minimal-change disease (MCD), mesangial proliferative glomerulonephritis (MesPGN), focal segmental glomerulosclerosis (FSGS), or other histopathologies [1].

Treating SRNS, which should be done under the care of a pediatric nephrologist, can be challenging because there is a paucity of strong evidence to inform SRNS treatment decisions due to the lack of large-scale randomized controlled trials (RCTs). Children with SRNS may be treated with immunosuppressive agents, such as cyclosporin, cyclophosphamide, or tacrolimus [3]. Remission rates obtained with combinations of cyclophosphamide and intravenous (i.v.) methylprednisolone have reached 50–60% in observational studies and individual treatment groups in RCTs [4–7]. Meanwhile, complete remission (CR) and partial remission (PR) rates with calcineurin inhibitors (cyclosporine and tacrolimus) have been in the range of 30–80% in observational studies and RCTs [8–10]. If there is a failure to achieve at least PR, SRNS prhogresses to end-stage kidney disease [11, 12].

In recent decades, several new lower-toxicity immunosuppressive medications have been introduced for the treatment of SRNS in children [13]. However, these new medications have been found to be less effective for prolonging remission after corticosteroid withdrawal than traditional immunosuppressant drugs. Because head-to-head comparison trials of these new agents with traditional ones have not been completed, however, there is not a consensus regarding which immunosuppressive drugs are most suitable for treating SRNS in children Pairwise meta-analyses evaluating the efficacy of new immunosuppressive medications have identified factors that may be associated with therapeutic efficacy and previous systematic reviews have suggested efficacy differences among nonsteroidal immunosuppressive medications [14–16]. However, these studies are inconclusive because they could not provide direct comparisons. Moreover, the extent to which efficacy and safety varies across potential SRNS drugs is unclear.

Here, we report a network meta-analysis in which nine nonsteroidal immunosuppressive agents were compared with respect to efficacy and safety in children being treated for SRNS. The aim of this work was to identify a preferable SRNS therapy drug in children.

## RESULTS

### Study characteristics and evidence network

**Figure 1:**
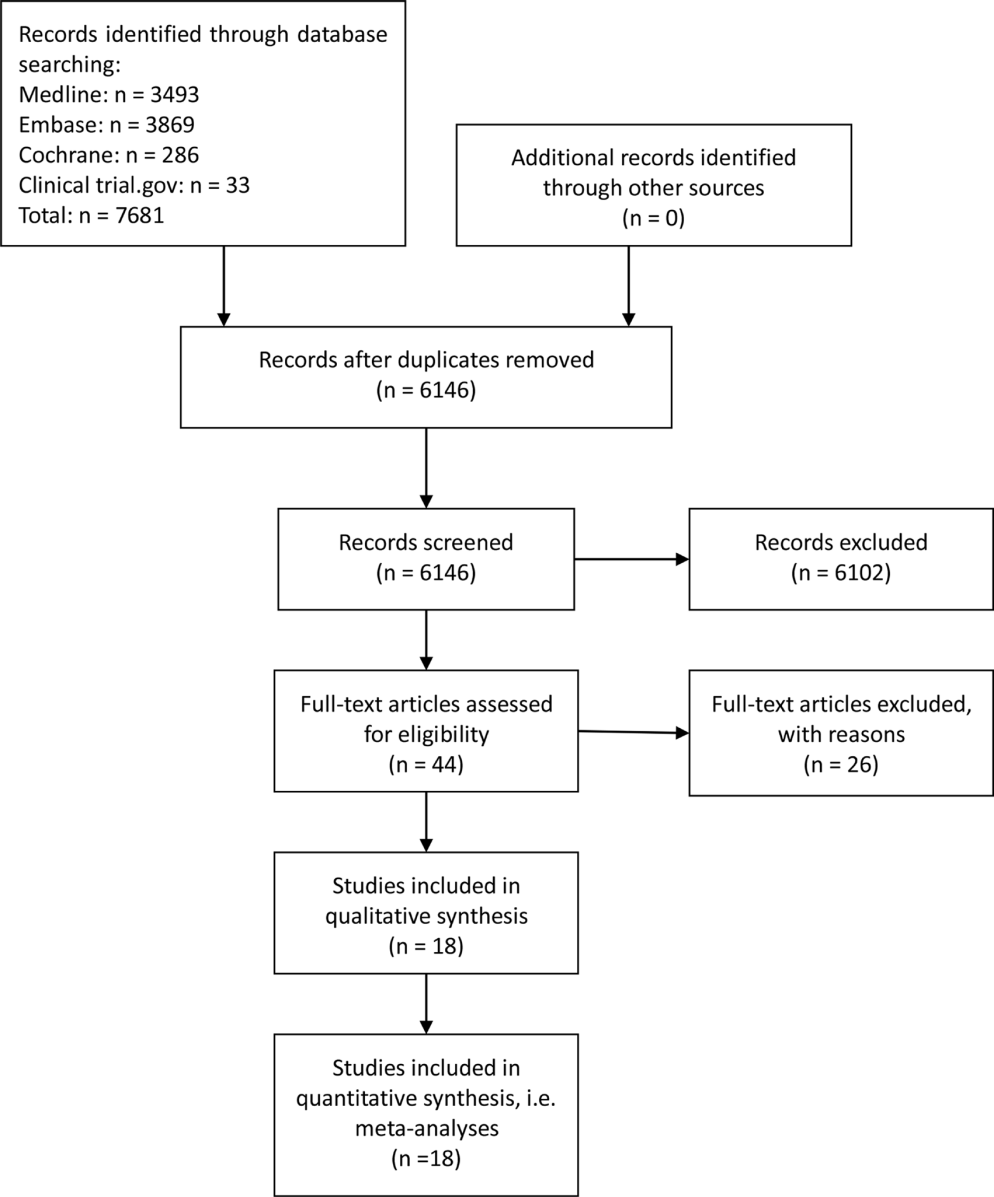
Flowchart of included studies

A total of 7,681 potentially relevant studies were retrieved, which included 6,146 non-repetitive potentially eligible articles. On the basis of our eligibility criteria (parallel RCTs whose subjects were children with initial SRNS and children with delayed SRNS and that were examining immunosuppressive medications), 6,102 articles were excluded during the title/abstract review process and, subsequently, 26 articles were excluded consequent to a full-text review. Finally, 18 articles published from 1970 to 2015 were included in our network meta-analysis. The 18 selected studies involved a total of 790 individuals who were assigned randomly to an immunosuppressive medication or placebo/nontreatment (P/NT) group. The trial selection process is summarized in Figure 1.

### Study characteristics

**Figure 2:**
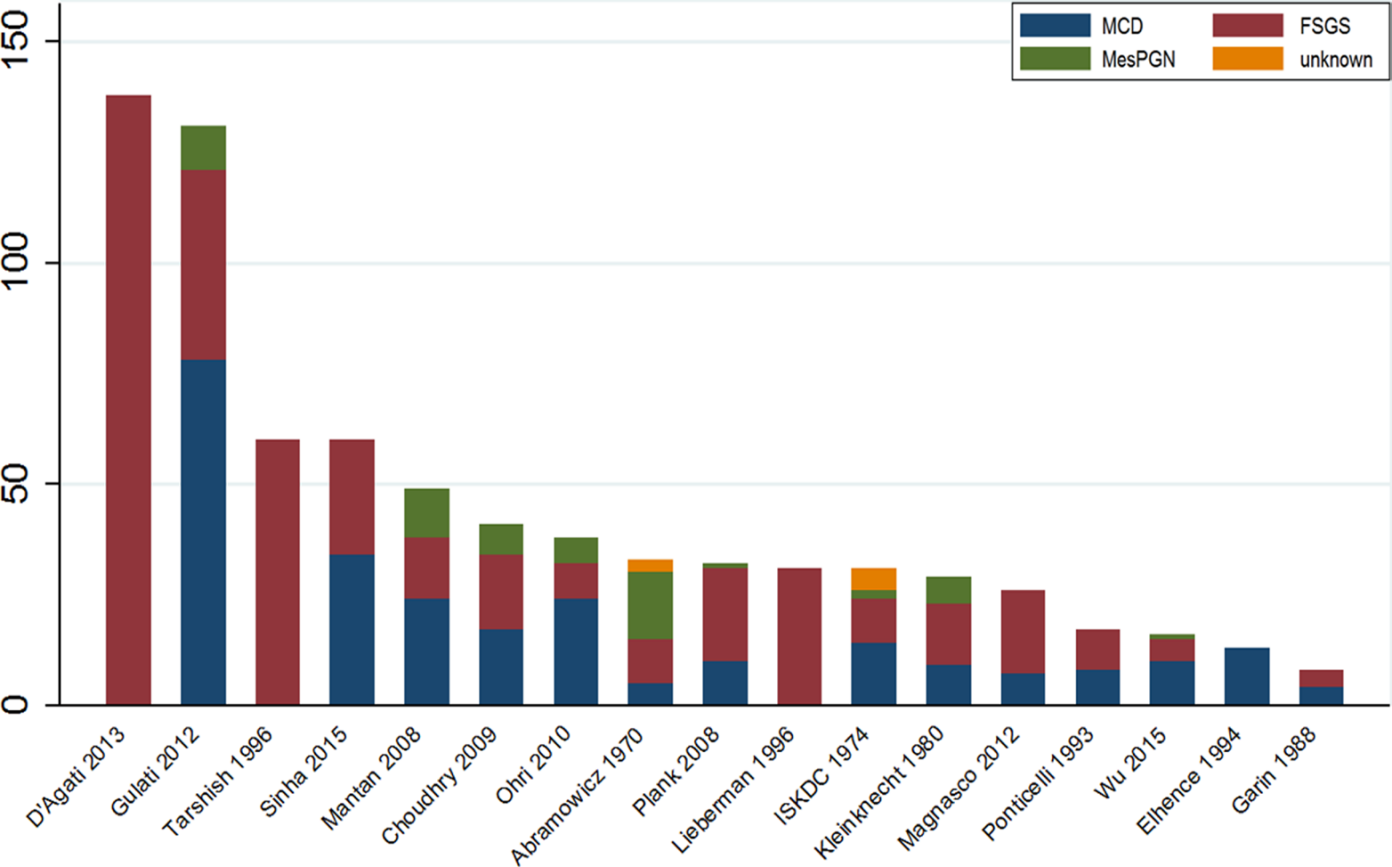
Distribution of histopathologic diagnoses in included RCTs

The characteristics of the 18 included trials are summarized in Table 1 [4, 6–9, 17–29]. Briefly, trial durations ranged from 3 months to 24 months and the enrolled patients ranged in age from 1 year to 18 years old. A majority (61%) of the participants were male. The distribution of histopathologic diagnoses in each trial are shown in Figure 2. Most (14/17, 82%) of the studies included patients with MCD, MesPGN, and FSGS, three studies included only patients with FSGS, and one study included only patients with MCD. Data from 790 individuals were included in the efficacy and safety analyses. The mean sample size was 44 individuals per group (range, 8–138). Most (17/18; 94%) of the studies had two arms; one study had three arms. Regarding study quality, 67% of the trials were outcome-blinded, 56% were allocation-concealed, 50% had incomplete outcome data, and 17% were patient-blinded. Generally, the risk of bias in the reviewed trials was medium (see Supplementary Figure 1).

**Table 1:** Descriptive characteristics of studies included in the meta-analysis

Study	Country	Study design	Time frame	Cases (N)	Age (y)	Sex (M/F)	Patients	Interventions	Study outcomes	Follow-up duration	Attrition (%)
Abramowicz 1970	International	Multicenter RCT	1967–1969	38	NR	NR	Initial SRNS	AZA vs P/NT	CR and PR at 90 d,	3 mos.	18%
Choudhry 2009	India	Single center RCT	2005–2007	41	3.5–6.0	25/16	Initial and late SRNS	TAC vs CSA	CR and PR at 12 mos., adverse effects	12 mos.	0%
D'Agati 2013	USA	Multicenter RCT	2004–2009	138	NR	73/65	Initial SRNS	CSA vs MMF	CR and PR at 12 mos., adverse effects	12 mos.	1%
Elhence 1994	India	Single center RCT	1990–1991	13	3–16	11/2	Initial and late SRNS	OCPA vs ICPA	CR at 6 mos., adverse effects	6 mos.	15%
Garin 1988	USA	Single center RCT	NR	8	3–18	6/2	SRNS	CSA vs P/NT	CR at 3 mos.	3 mos.	0%
Gulati 2012	India	Multicenter RCT	2008–2010	131	2–16	86/45	Initial and late SRNS	TAC vs ICPA	CR and PR at 12 mos., adverse effects	12 mos.	5%
ISKDC 1974	International	Multicenter RCT	1970–1972	31	NR	NR	Initial SRNS	OCPA vs P/NT	CR and PR at 12 mos., adverse effects	24 mos.	0%
Kleinknecht 1980	France	Single center RCT	NR	30	NR	NR	SRNS	CHL vs P/NT	CR at 6 mos.	6 mos.	0%
Lieberman 1996	USA	Multicenter RCT	NR	31	7–16	21/9	Initial SRNS	CSA vs P/NT	CR and PR at 6 mos., adverse effects	6 mos.	23%
Magnasco 2012	Italy	Single center RCT	2007–2010	31	< 16	19/12	Initial and late SRNS	RTCA vs P/NT	CR at 12 mos., adverse effects	12 mos.	0%
Mantan 2008	India	Single center RCT	2001–2003	52	1–18	35/17	Initial and late SRNS	ICPA vs OCPA	CR and PR at 6 mos., adverse effects	18 mos.	6%
Ohri 2010	India	Single center RCT	NR	35	1–12	17/18	Initial SRNS	ICPA vs OCPA	CR and PR at 6 mos., adverse effects	6 mos.	0%
Plank 2008	International	Multicenter RCT	2001–2004	32	1–13	19/13	Initial SRNS	CSA vs CPA	CR and PR at 3 mos., adverse effects	12 mos.	33%
Ponticelli 1993	Italy	Multicenter RCT	NR	20	2–18	NR	Initial SRNS	CSA vs P/NT	CR and PR at 12 mos., adverse effects	12 mos.	15%
Sinha 2015	India	Multicenter RCT	NR	60	1–18	NR	SRNS	TAC vs MMF	Complete or PR at 12 mos.	12 mos.	0.0%
Tarshish 1996	International	Multicenter RCT	NR	60	1–16	NR	Initial SRNS	OCPA vs P/NT	CR and PR at 6 mos., adverse effects	12 mos.	11%
Valverde 2010	Mexico	Single center RCT	NR	17	1–18	NR	SRNS	CSA vs TAC	CR and PR at 12 mos., adverse effects	12 mos.	0%
Wu 2015	China	Single center RCT	2008–2012	18	2–18	11/7	SRNS	CPA vs MMF vs LEF	CR at 6 mos.	12 mos.	18%

Notes: SRNS, steroid-resistant nephrotic syndrome; NR, not reported; RCT, randomized controlled trial; CSA, cyclosporin; ICPA, intravenous cyclophosphamide; TAC, tacrolimus, MMF, mycophenolate mofetil; OCPA, oral cyclophosphamide; LEF, leflunomide; CHL, chlorambucil; AZA, azathioprine; RTCA, rituximab-cyclosporin dual therapy; P/NT, placebo/nontreatment; CR, complete response; PR, partial response.

### Evidence network

**Figure 3:**
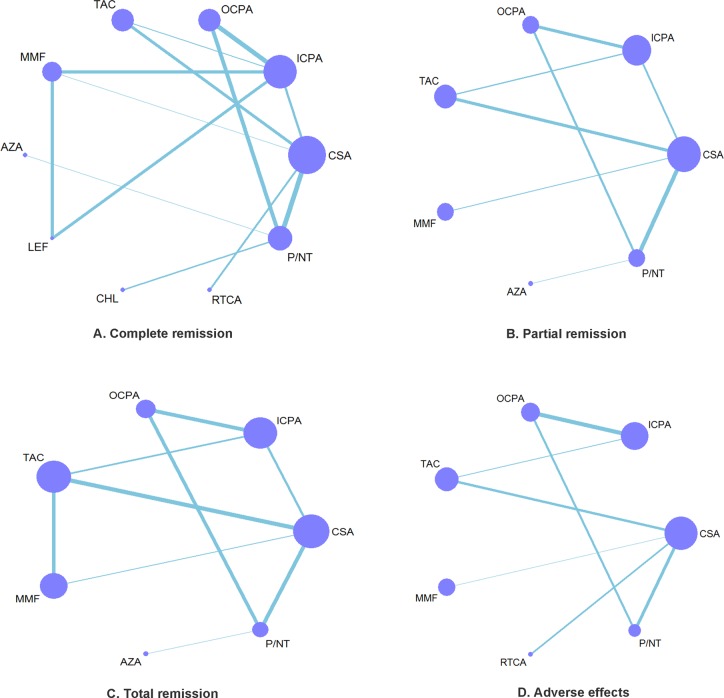
Network of eligible efficacy and safety comparisons (**A**–**D**) The thickness of the lines reflects the number of studies being compared, and node size reflects the number of individuals treated with each pharmacotherapy.

Our efficacy analyses included 730 patients with a CR in 17 trials examining a total of ten treatments (Figure 3A), 569 patients with a PR in 12 trials examining a total of seven treatments (Figure 3B), and 605 TR patients in 12 trials examining a total of seven treatments (Figure 3C). Our safety analyses based on the incidence of ASEs included 730 patients in 11 trials examining a total of seven treatments (Figure 3D). Ultimately, the following nine treatments were analyzed relative to P/NT in the present meta-analysis: cyclosporin (7 trials), i.v. cyclophosphamide (3 trials), tacrolimus (4 trials), MMF (3 trials), oral cyclophosphamide (5 trials), leflunomide (1 trial), chlorambucil (1 trial), azathioprine (1 trials), and rituximab-cyclosporin dual therapy (1 trial).

### Conventional meta-analysis of individual medications

**Figure 4:**
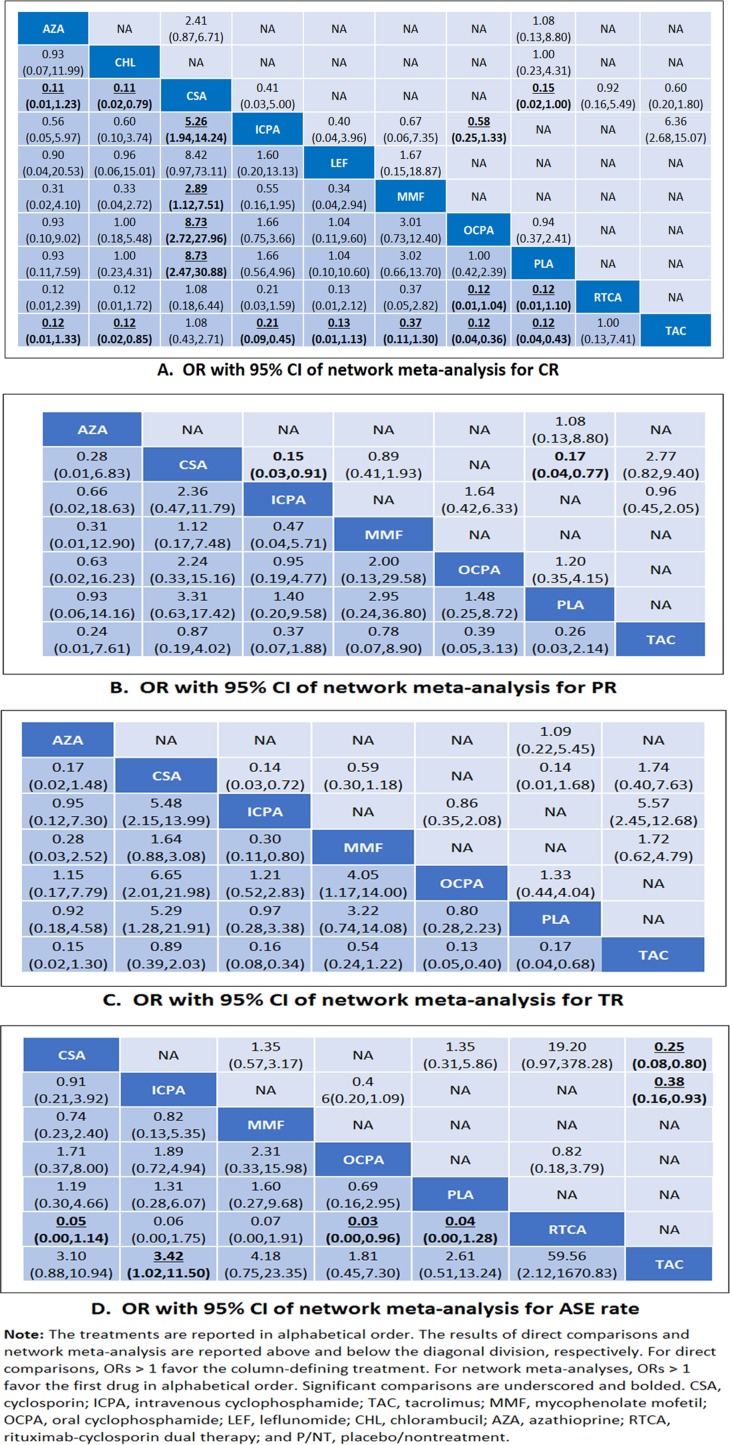
Comparison of efficacy across drugs OR with 95% CI of network meta-analysis for CR (**A**), PR (**B**), TR (**C**) and ASE (**D**).

The efficacy and safety analysis results obtained for individual immunosuppressive medications as determined by direct pairwise meta-analyses are shown in Figure 4. Relative to P/NT, cyclosporin was found to have significantly better efficacy for achieving both CR and PR (both, I^2^ = 0%). Cyclosporin also showed better efficacy than i.v. cyclophosphamide for both PR and TR outcomes (both I^2^ = 0%), as well as greater efficacy than MMF for TR (I^2^ = 0%), though in the latter case the benefits were less clear (95% CI for OR slightly more than 1). Additionally, tacrolimus was more efficacious than i.v. cyclophosphamide for TR (I^2^ = 0%) and i.v. cyclophosphamide was more efficacious than oral cyclophosphamide for CR (I^2^ = 37.8%).

In terms of ASEs, oral cyclophosphamide was found to be safer than i.v. cyclophosphamide (95% CI for OR slightly more than 1) and tacrolimus was found to be safer than both cyclosporin and i.v. cyclophosphamide (I^2^ = 15.1%). There were no significant differences in safety revealed by other direct comparisons between immunosuppressants. However, it should be noted that the 95% CIs obtained for most of the comparisons were reflective of either high or no heterogeneity due to the small number of studies included in the pairwise comparisons; overall, heterogeneity was moderate.

### Network meta-analysis of individual medications

Our network meta-analysis results for immunosuppressive medications, active comparators, and P/NT are presented in Figure 4. Tacrolimus was found to be more efficacious for achieving CR than i.v. cyclophosphamide, MMF, oral cyclophosphamide, leflunomide, chlorambucil, and azathioprine, as well as P/NT. Tacrolimus was also found to be more efficacious for achieving TR than i.v. cyclophosphamide, oral cyclophosphamide, azathioprine, and P/NT. Meanwhile, cyclosporin therapy was associated with a better CR rate than i.v. cyclophosphamide, MMF, oral cyclophosphamide, chlorambucil, azathioprine, or P/NT. Cyclosporin was more likely to yield more TR outcomes than i.v. cyclophosphamide, oral cyclophosphamide, or P/NT. Additionally, rituximab-cyclosporin dual therapy was more efficacious for obtaining CR than oral cyclophosphamide or P/NT, and MMF more efficacious in terms of TR than either i.v. cyclophosphamide or oral cyclophosphamide. However, in terms of safety, tacrolimus use was less likely to result in ASEs than i.v. cyclophosphamide or rituximab-cyclosporin dual therapy. Meanwhile, rituximab-cyclosporin dual therapy was associated with a greater likelihood of ASEs than oral cyclophosphamide, cyclosporin, or P/NT (95% CI for OR slightly more than 1).

### Ranking of medications

**Figure 5:**
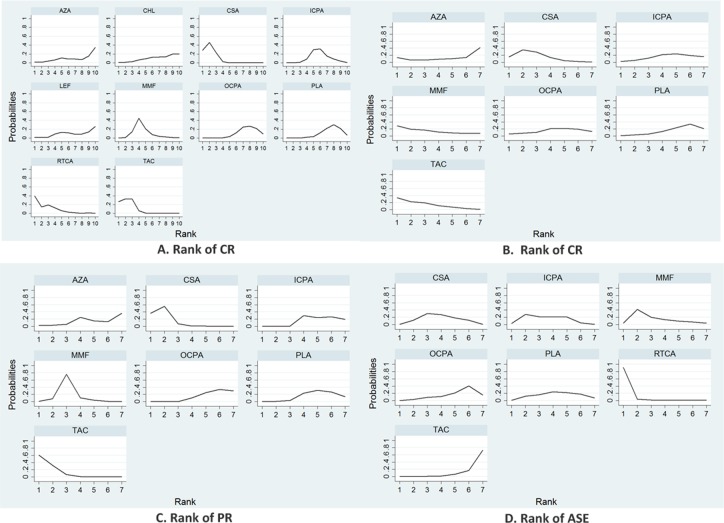
Efficacy and safety outcome rankings CR (**A**), PR (**B**), TR (**C**) and ASE (**D**) rankings reflect the probability of being the best, second best, etc., treatment among the treatments compared.

The relative efficacy and safety rankings of the interventions are shown in Figure 5. Cyclosporin, tacrolimus, rituximab-cyclosporin dual therapy, and MMF were among the most efficacious treatments for achieving CR (Figure 5A). The cumulative probabilities of CR for the examined medications were: cyclosporin (88.7%), tacrolimus (86.4%), rituximab-cyclosporin (82.8%), MMF (59.8%), i.v. cyclophosphamide (44.8%), leflunomide (31.5%), chlorambucil (28.6%), azathioprine (28.6%), P/NT (24.5%), and oral cyclophosphamide (24.2%). Tacrolimus, cyclosporin, and MMF were the most efficacious treatments for achieving of PR (Figure 5B), and the cumulative probabilities of the analyzed pharamcotherapies being the most efficacious medication were: tacrolimus (74.1%), cyclosporin (71.7%), MMF (65.9%), oral cyclophosphamide (41.1%), i.v. cyclophosphamide (37.6%), azathioprine (33.5%), and P/NT (26.1%). Finally, for TR, tacrolimus, cyclosporin, and MMF were the most efficacious treatments (Figure 5C), and the cumulative probabilities of each treatment being the most efficacious medication were: tacrolimus (91.5%), cyclosporin (87.8%), MMF (65.7%), P/NT (29.2%), i.v. cyclophosphamide (28.1%), azathioprine (28.1%) and oral cyclophosphamide (19.6%). In terms of safety, rituximab-cyclosporin dual therapy was the treatment that was most likely to produce ASEs (Figure 5D), and the cumulative probabilities of being the most adverse medication were: rituximab-cyclosporin (96.8%), MMF (62.8%), intravenous cyclophosphamide (58.3%), cyclosporin (52.4%), P/NT (44.8%), oral cyclophosphamide (27.8%), and tacrolimus (7.3%).

### Inconsistency and publication bias

**Figure 6:**
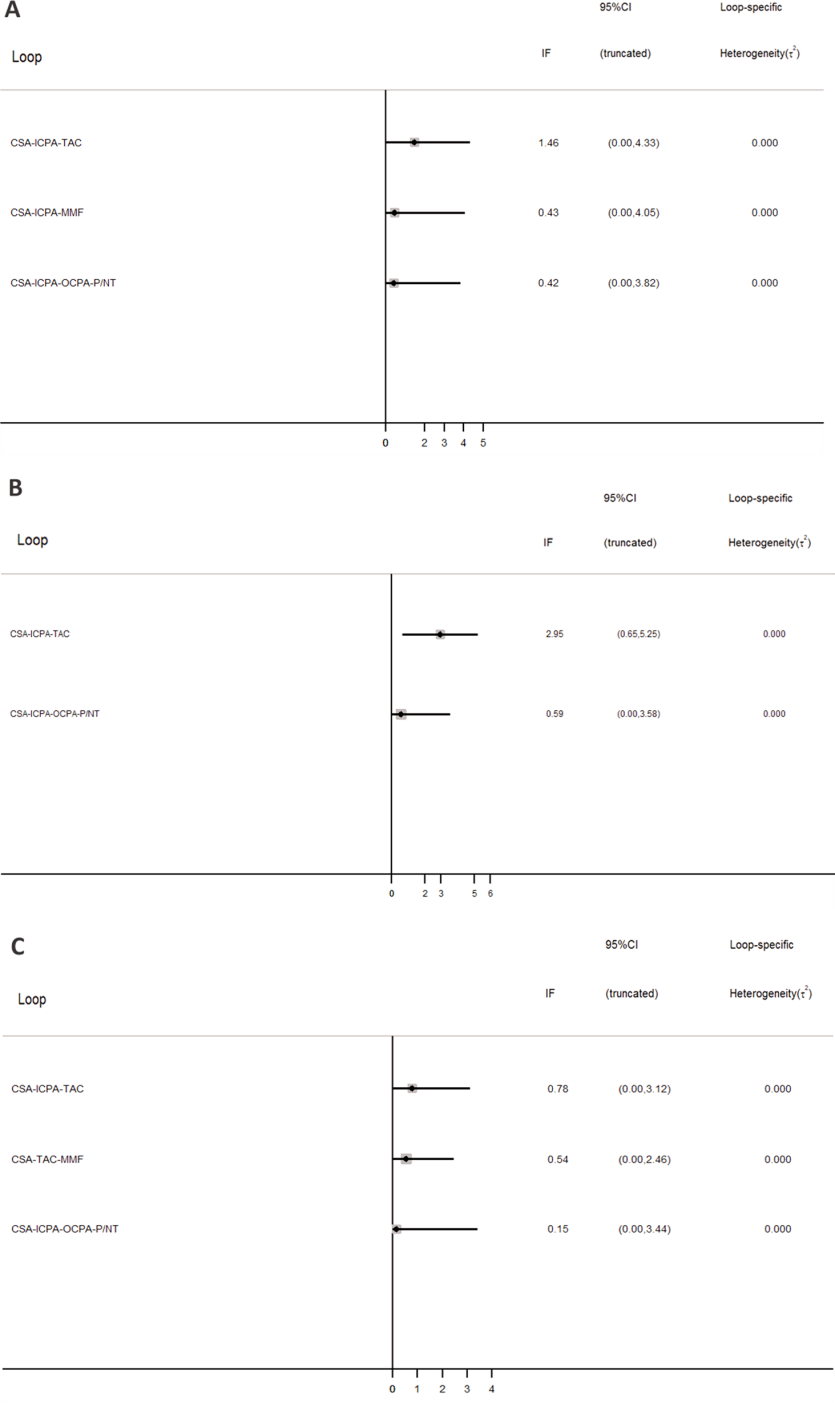
Inconsistency in closed loops at CR (**A**) PR (**B**) and TR (**C**). Graph shows estimates of differences between direct and indirect estimates as represented by 95% CIs.

**Figure 7:**
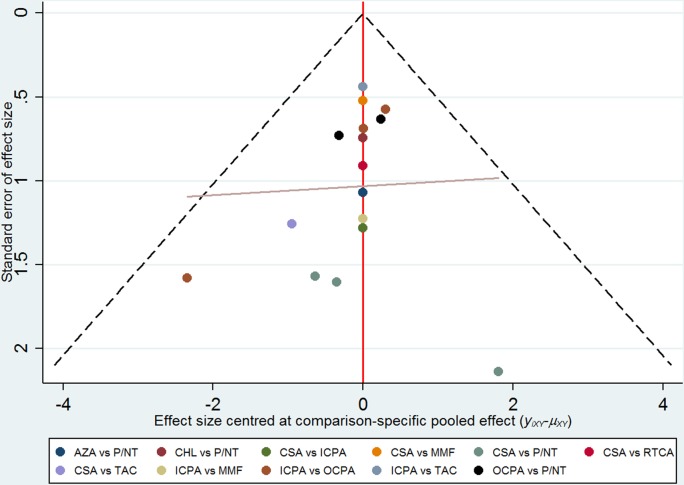
Summary of comparison-adjusted funnel plot results for CR rate

Inconsistency between direct and indirect comparisons of recurrence rates was low (Figure 6). Most loops (networks of three or four comparisons that arise when collating studies involving different treatments) were consistent, with the 95% CIs for the inconsistency factor (including 0) indicating similar effect estimations for direct and indirect comparisons. Hence, the network meta-analysis results can be considered robust. Comparison-adjusted funnel plots for CR outcomes show no evidence of asymmetry (Figure 7).

## DISCUSSION

In the present meta-analysis of 18 trials, with 790 individuals diagnosed with SRNS assigned randomly to one of nine immunosuppressive medication groups or a P/NT group, we found that tacrolimus was more efficacious for achieving CR or TR than i.v. cyclophosphamide, MMF, oral cyclophosphamide, leflunomide, chlorambucil, azathioprine, and P/NT, with a lower ASE risk than i.v. cyclophosphamide or rituximab-cyclosporin dual therapy. Cyclosporin also fared well, being associated with greater likelihood of CR or TR than intravenous cyclophosphamide, MMF, oral cyclophosphamide, chlorambucil, azathioprine, or P/NT. Hence, of the nine immunosuppressive pharmacotherapies analyzed, tacrolimus and cyclosporin emerged as the two most efficacious agents while maintaining relatively low ASE risk levels. A practical implication of our results is that tacrolimus and cyclosporin should be favored as first-line treatments for pediatric patients experiencing SRNS owing to their high efficacy and generally good, albeit not superior, safety. Given that tacrolimus had a similar efficacy but lower ASE likelihood than cyclosporin, further well designed RCTs are needed to evaluate the relative benefits and harms of tacrolimus versus cyclosporin for the treatment of SRNS in children.

Although MMF ranked favorably in efficacy and safety, the MMF data lacked power in the network meta-analysis indicating that there is a need for additional studies of the efficacy and safety of MMF in children with SRNS. The efficacy and safety outcomes for cyclophosphamide therapies were mediocre. Notwithstanding, it is worth noting that our analysis showed greater efficacy, but lesser safety, for i.v. cyclophosphamide relative to oral cyclophosphamide. Hence, the present results do not support the use of cyclophosphamide as a first-line treatment for SRNS. Finally, although rituximab-cyclosporin dual therapy was found to have a somewhat favorable CR outcome likelihood, especially compared with oral cyclophosphamide, it had the poorest safety outcome of all of the regimens examined.

Our findings, which indicate clinically important differences in efficacy and safety among the examined drugs, provide reference information that can be used in immunosuppressive medication selection for treatment of SRNS in pediatric patients. Previous conventional pairwise meta-analyses of the efficacy of immunosuppressive medications for SRNS in children were inconclusive due to limitations in treatment effect information and failures to demonstrate clear relative efficacy benefits of particular drugs [15, 16, 30, 31]. Notwithstanding, a previous systematic review contributed by Hodson et al. indicated that calcineurin inhibitors yield better rates of CR or PR than P/NT or cyclophosphamide in children with SRNS [30].

The present study provides much needed direct comparisons between immunosuppressive agents in patients recovering from primary NS. The 2015 clinical guidelines for pediatric idiopathic NS recommended cyclosporine as a first-line treatment for SRNS (recommendation grade A). The guidelines suggested that tacrolimus be considered as a treatment option for patients with SRNS when cyclosporine is counter-indicated due to cosmetic side effects (recommendation grade C1), and that cyclophosphamide not be considered for induction therapy in children with SRNS (recommendation grade C2) [32].

The most problematic ASE of long-term cyclosporine use is chronic nephrotoxicity, an increased risk for which has been associated with cyclosporine treatment for ≥ 2 years [33–35]. The number of serious ASEs of immunosuppressive agents reported here may be an underestimate because the analyzed studies were not designed primarily to evaluate harm. Additionally, because we did not exclude trials with combined corticosteroid regimens, our findings related to immunosuppressive agents cannot be considered independent of potential corticosteroid effects. Our results should not be generalized to patients who exhibit corticosteroid dependence because we did not include trials with that patient population.

The results of this analysis apply to treatment periods of 2 years or less. Longer term clinical efficacy and safety beyond 2 years may differ substantially from outcomes recorded within 2 years [35]. Additionally, the quality of our analysis may be limited by the quality of the original data. Many of the studies included in our review did not report adequate information on allocation concealment and randomization, which could influence the overall validity of the data [36]. Only 17% of the examined studies had low performance risk. We did not conduct publication bias or subgroup analyses due to the small numbers of studies examining each medication. The small number and small sample sizes of the included studies could also be of concern for the generalizability of our results. Finally, none of the included studies addressed fertility-related complications of alkylating agent therapy.

This study focused on examining RCTs of available immunosuppressive agents in pediatric SRNS patients. Recently, ongoing or completed small sample case series of new biologics—including anti-CD20 ofatumumab, abatacept, adalimumab, fresolimumab, and saquinavir which target immune cell subsets or activation pathways selectively—have spurred a new direction of hypothesis-driven therapies that may improve outcomes of children with kidney disease [37, 38]. Further research should be conducted to determine the benefits and risks of these therapies in children with SRNS.

In conclusion, on the basis of all available direct and indirect evidence, our results suggest that tacrolimus and cyclosporine are preferable first-line medications for initial SRNS in children. MMF may be an acceptable option for patients with SRNS. Further studies are needed to evaluate the relative benefits and harms of tacrolimus versus cyclosporin for pediatric SRNS treatment. Additional information about the safety and efficacy of MMF in children with SRNS is also needed.

## MATERIALS AND METHODS

### Identification of trials

In preparation for this network meta-analysis, we drafted a study protocol and published it on the PROSPERO website (CRD42017062564). Clinical trials comparing at least two different treatments were searched in MEDLINE (1950 to January 2017), the Cochrane Central Register of Controlled Trials (CENTRAL, The Cochrane Library, Issue 7, 2017), and EMBASE (1974 to January 2017) with the following search terms: “immunosuppressive agents” or “alkylating agents” or “azathioprine” or “cyclosporine” or “cyclophosphamide” or “mycophenolic acid” or “rituximab” or “chlorambucil” or “levamisole” or “tacrolimus”; and “nephrotic syndrome” or “minimal change nephrotic syndrome” or “glomerulonephritis membranoproliferative” or “focal segment glomerulosclerosis” or “membranoproliferative glomerulonephritis”. The search results were restricted to articles reporting studies involving children. Additionally, the reference lists of all included publications and relevant reviews were screened and ClinicalTrials.gov was searched for trials in progress.

### Selection criteria

Parallel RCTs in which children with initial SRNS and children with delayed SRNS were the subjects and comprehensive comparisons of any of the following agents were included: cyclophosphamide, cyclosporine, tacrolimus, mycophenolate mofetil (MMF), azathioprine, chlorambucil, rituximab, or levamisole. The experimental interventions could be compared to a placebo/nontreatment (P/NT) and/or another immunosuppressive medication. SRNS was defined as persistence of proteinuria > 3+ on a dipstick test, urinary protein-creatinine ratio (UP/C) > 0.2 g/mmol or 40 mg/m^2^/h after four weeks or more of daily corticosteroid use. Trials involving patients experiencing steroid-sensitive nephrotic syndrome, congenital nephrotic syndrome, and other kidney or systemic forms were excluded. Trials for which only abstracts were published (with no additional data available from other sources) were also excluded. No language restrictions were applied.

### Outcome measures

The primary efficacy outcomes were number in CR, number in PR, and total number in remission (TR). The primary safety outcome was the incidence of adverse secondary effects (ASEs). We defined CR as edema-free and urine protein was < 1+ on dipstick tests, urinary UP/C < 0.02 g/mmol or 4 mg/m^2^/h for three or more consecutive days. We defined PR as proteinuria < 2+, urinary UP/C < 0.2 g/mmol or 40 mg/m^2^/h. TR was defined as the total number in remission, including patients in CR or PR. We defined ASEs as sequelae occurring within the initial posttreatment period (determined by each study's authors).

### Data extraction and risk of bias assessment

Two investigators (SL and LT) reviewed all of the abstracts and references, evaluated the integrity of data abstraction, and rated the quality of included studies independently. We sent emails to the authors of articles with incomplete information and asked them to provide supplemental data. Methodological quality and risk of bias were assessed with the Cochrane risk of bias tool.

### Statistical analysis

All data analyses were conducted in Stata, version 14 software (Stata Corp, College Station, TX, USA). First, we performed pairwise random-effects meta-analyses using the Knapp-Hartung method with the metan command, from which we report odds ratios (ORs) and 95% confidence intervals (CIs) [39]. When a zero event was reported in a trial, the Haldane method was used to add 0.5 to each arm. We conducted traditional pairwise comparisons using the DerSimonian-Laird random effects model, which discerns and anchors trials as a sample of all potential trials. The I^2^ statistic was obtained as an index of heterogeneity.

Second, we conducted a network meta-analyses within a frequentist framework using the Stata network suite [40]. A multivariate random-effects meta-analysis (mvmeta command) was performed with the assumption that heterogeneity variance was consistent across treatment contrasts. We used the netleague command to report relative treatment effects for all pairwise comparisons estimated by the network meta-analysis. *P* < 0.05 was considered significant. We looked at a plausible range for population difference magnitude. We used the network rank option to evaluate the probability that each drug could be the most (or second most, third most, etc.) efficacious treatment. The surface under the cumulative ranking curve (SUCRA) was determined as an estimation of the ranking probability for each medication, and the resultant SUCRA estimates were used to rank the treatments in a hierarchy [41]. We ranked the medications’ safety with the same method. Within the networks, we assessed consistency between direct and indirect evidence using the design-by-treatment interaction model [42]. A loop-specific approach was applied to detect local inconsistencies within network meta-analysis models if information was sufficiently similar across sources to be combined. Difference (inconsistency factor) between direct and indirect estimations for a specific comparison was calculated with 95% CIs as a measure of within-loop inconsistency [43]. Inconsistency was defined as disagreement between direct and indirect evidence with a 95% CI excluding 0. Publication bias was estimated by comparison-adjusted funnel plots.

## SUPPLEMENTARY MATERIALS FIGURE



## Abbreviations

SRNS: steroid-resistant nephrotic syndrome
RCT: randomized controlled trial
I.v.: intravenous
MCD: minimal-change disease
MesPGN: mesangial proliferative glomerulonephritis
FSGS: focal segmental glomerulosclerosis
P/NT: placebo/nontreatment
MMF: mycophenolate mofetil
CR: complete remission
PR: partial remission
TR: total number in remission
OR: odds ratio
NS: nephrotic syndrome
CI: confidence interval
UP/C: protein-creatinine ratio
ASE: adverse secondary effect
SUCRA: surface under the cumulative ranking curve.
