# Supplementary material for: Efficacy and safety of immunosuppressive medications for steroid-resistant nephrotic syndrome in children: a systematic review and network meta-analysis
Source: Oncotarget. 2017 Aug 21;8(42):73050–62. doi: 10.18632/oncotarget.20377 (PMC5641191; doi:10.18632/oncotarget.20377)
Supplement: Supplementary file 1 [file oncotarget-08-73050-s001.pdf]

Efficacy and safety of immunosuppressive medications for steroid-resistant nephrotic syndrome in children: a systematic review and network meta-analysis

SUPPLEMENTARY MATERIALS

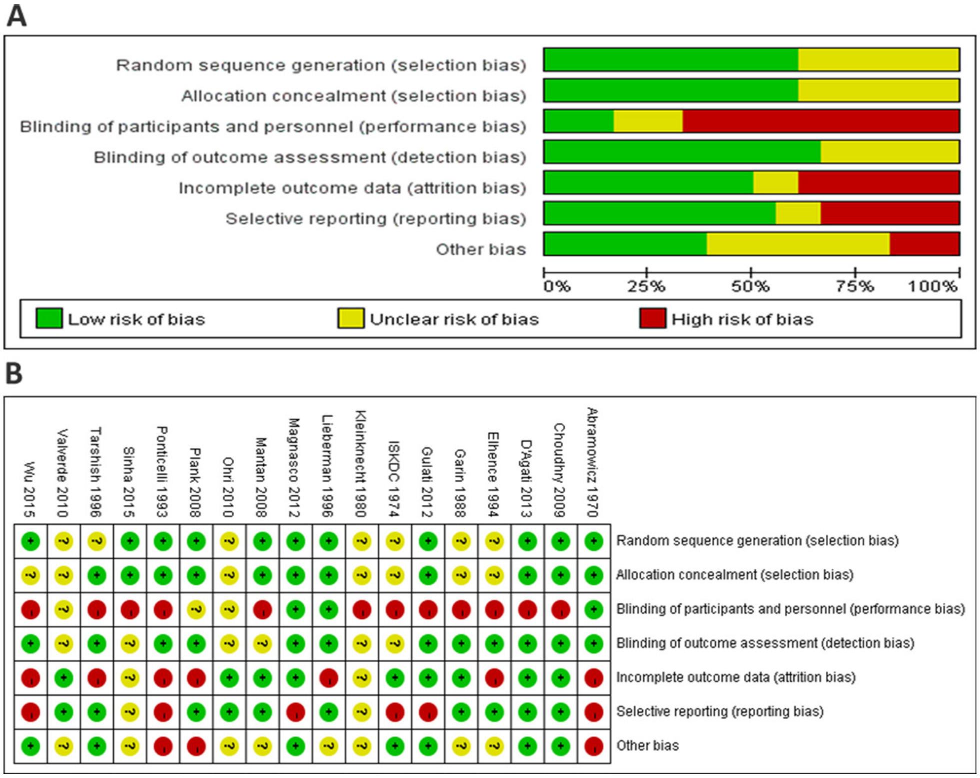

Supplementary Figure 1: Risk of bias. (**A**) Summary of bias across all included studies. (**B**) Characteristics of bias risk for each of the individual included studies.
